# H_2_ Adsorbed Site-to-Site Electronic Delocalization within IRMOF-1: Understanding Non-Negligible Interactions at High Pressure

**DOI:** 10.3390/ma9070578

**Published:** 2016-07-15

**Authors:** Jian Wu, Mustafa U. Kucukkal, Aurora E. Clark

**Affiliations:** Department of Chemistry, Washington State University, Pullman, WA 99164, USA; jian.wu@wsu.edu (J.W.); m.kucukkal@wsu.edu (M.U.K.)

**Keywords:** IRMOF-1, H_2_ adsorption, MOF-5, high-pressure interactions

## Abstract

Isoreticular metal organic frameworks (IRMOFs) have shown high uptake capabilities for storage of H_2_ (11.5 wt % at 77 K and 170 bar). A significant literature has employed fragment models and a single adsorbed H_2_ to identify adsorption sites within IRMOFs, as well as the necessary adsorbate–adsorbent interactions needed to reach sufficient adsorption enthalpy for practical usage, however at high pressures it remains to be seen if H_2_···H_2_ intermolecular interactions may influence the energetics. This study focuses upon IRMOF-1 (also known as MOF-5), and examines the individual H_2_ stabilization energies at different sites using Möller–Plesset perturbation theory and density functional theory alongside chemical models that consist of isolated fragment models and a cubic super cell cluster consisting of both the face- and edge-cube’s of IRMOF-1. Optimization of twenty stable configurations of singly adsorbed H_2_ in the super-cell cluster is observed to be essential to obtain energy ordering of the five primary sites consistent with experiment and prior benchmark calculations (α >> β > γ > δ ≈ ε). To examine site-to-site interactions that may occur in the high-pressure regime, 64 co-adsorbed H_2_ within a super-cell cluster have been studied (a theoretical maximum of all adsorption sites, 14 wt %). There, delocalization and/or charge transfer of electrons is observed from the σ orbitals of the H_2_ bound at the γ positions into the σ* orbitals of H_2_ bound at the α sites leads to stabilization of the interaction of H_2_ at the γ, by 1.4 kJ/mol, respectively (using M06-2X/LANL2DZ). This effect has been confirmed to be charge transfer, and not a manifestation of enhanced dispersion at high loading, through natural bond order (NBO) analysis and by comparisons of the square of off-diagonal NBO Fock matrix elements for both density functionals that account for dispersion interactions and Hartree–Fock calculations that ignore dispersion.

## 1. Introduction

**Figure 1:**
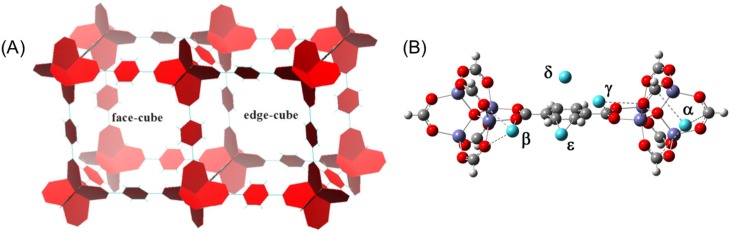
(**A**) The face-cube cell and edge-cube cell of IRMOF-1; (**B**) The five primary adsorption sites, α, β, γ, δ and ε, of hydrogen in IRMOF-1 (denoted by cyan spheres).

The isoreticular series of metal organic frameworks (IRMOFs) are the first metal-organic framework species that have shown the potential for high uptake of H_2_ [1]. IRMOF-1 is representative of the basic structural and reactive properties for this series [2], having a highly porous cubic framework composed of (Zn_4_O)^6+^ clusters as nodes and 1,4-benzenedicarboxylate (BDC) groups as linkers. IRMOF-1, also known as MOF-5, has exhibited exceptionally high H_2_ uptake of 7.1 wt % at ~40 bar and 77 K, ~10.3 wt % at 30 K at ~4 bar and a maximum capacity of 11.5 wt % at 3.5 K (or ~170 bar at 77 K) [3,4]. However, at room temperature, the H_2_ storage capacity is significantly diminished, between 0.2 and 0.45 wt % [5,6,7,8], because the known H_2_ binding sites have interaction energies <8 kJ/mol [9]. Much can be learned from the IRMOF series and a detailed understanding of H_2_ sorption behavior that accounts for both the molecular and collective interactions is a necessary prerequisite for the rational design of porous media for high capacity H_2_ storage. Complementary computational and experimental studies have focused primarily upon identification of the sorption sites, configurations that H_2_ can adopt during sorption, and the interaction strength between H_2_ and IRMOF-1 at each site. Difference-Fourier analysis of neutron powder diffraction data, in addition to quantum mechanical (QM) and molecular dynamics studies of H_2_ adsorbed IRMOF-1 have identified five sites (α, β, γ, δ and ε) that are associated with two different cubic unit cells within the crystal, denoted “face-cube” and “edge-cube” (Figure 1) [10,11,12,13,14,15,16]. In the face-cube cell, the planes of twelve BDC linkers are oriented to the center of the unit cell, while in the edge-cube cell, the edges of twelve BDC linkers are oriented to the center. In the bulk system, one cube always connects to six other cubes by sharing four (Zn_4_O)^6+^ clusters and four BDC linkers.

Literature reports on H_2_ adsorption to IRMOF-1 are dominated by static quantum mechanical studies that can be broadly divided into two groups based upon whether the chemical model employed consists of fragments of the MOF building blocks, or a representation of the crystal. Table S1 in Supplementary Materials presents a compilation of computational methods, basis sets, and other technical parameters utilized within the majority of literature reports. Fragment based chemical models are amenable to highly correlated electronic structure methods (coupled cluster implementations, for example Coupled Cluster Singles Doubles and perturbative Triples (CCSD(T)), or Møller–Plesset 2nd order perturbation theory, MP2) that can accurately capture the weak dispersion forces that dominate the H_2_ interaction with IRMOF-1. In this context, MP2 has predicted a stabilization energy between H_2_ and H_2_-BDC-H_2_ (i.e., each oxygen atom of the BDC molecule is saturated by a hydrogen atom) of 5.27 kJ/mol in the basis set limit which is nearly 5 kJ/mol larger than the basis set extrapolated stabilization energy between H_2_ and benzene [12]. The H_2_ interaction with the Zn_4_O unit has been studied experimentally [5]. The H_2_ interaction with the Zn_4_O and BDC at various sites have also been studied computationally using both a two-body potential energy as well as QM methods with various basis sets [9,12,13,14,17,18]. The roles of dispersion, basis set superposition error (BSSE), complete basis set extrapolation, and the zero-point vibrational correction in H_2_ adsorption have also been discussed [9]. Recently, the specific adsorbate–adsorbent interactions were studied so that sufficient adsorption enthalpy for practical usage can be created [19]. The adsorption enthalpy was found to improve with charge transfer to the H_2_ from the adsorbent, as well as with the ability of the adsorbent to induce polarization of the H_2_ (particularly at linker sites). Density functional theory (DFT) with functionals that account for dispersion in combination with energy decomposition analysis (EDA) were utilized in this study.

In addition to the fragment models, a more limited suite of studies have used DFT in combination with Gaussian basis sets to optimize singly adsorbed H_2_ configurations within rigid non-periodic cells [12,13,17]. Plane wave calculations have generally optimized the unit cell using various numbers of atoms with periodic boundary conditions and then study H_2_ sorption via single point calculations or potential energy surface scans [9,11,20,21]. Previous studies using increasingly large IRMOF fragments have indicated that the charge distributions of the organic linkers are altered when embedded in a unit cell, in turn affecting the H_2_ interaction energies [22], though this has never been quantified. Large periodic cells, or even large non-periodic clusters further benefit from the ability to examine the H_2_ stabilization energy in the presence of other sorbed H_2_ and to study whether intermolecular adsorbate–adsorbate interactions may in turn alter the energetics of adsorption at high loading.

The current work first benchmarks the performance of the recently developed M06-2X [23] and ωB97XD [24] density functionals that account for dispersion interactions using a fragment based cluster model to study the interaction of H_2_ with each of the five primary adsorption sites in IRMOF-1. The hydrogen configurations are then explored in a super-cell cluster (non-periodic) that consists of 508 atoms (both face-cube and edge-cube) that have been optimized as a function of H_2_ loading. Natural Bond Order (NBO) analysis [25] is used to analyze the intermolecular H_2_···H_2_ interactions at optimized configurations at high loading (64 H_2_ in the super-cell cluster), whereupon it has been found that delocalization or charge transfer of electrons from the σ orbitals of the H_2_ bound at the γ positions into the σ* orbitals of H_2_ bound at the α sites. This leads to stabilization of the interaction of H_2_ at the γ site. This effect has been confirmed to be charge transfer, and not a manifestation of enhanced dispersion at high loading, through NBO analysis along the potential energy surface of the donating H_2_ to the center of the super-cell cluster using both M06-2X and Hartree–Fock (HF), and by comparisons of the square of off-diagonal NBO Fock matrix element with respect to the distance for both methods. This cooperative stabilization has implications for understanding H_2_ sorption at high pressures.

## 2. Computational Methods

**Figure 2:**
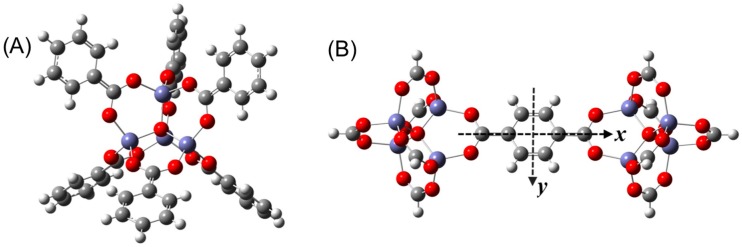
(**A**) The model frag1 of IRMOF-1; (**B**) The model frag2 of IRMOF-1.

The data herein only considers the change in electronic energy during H_2_ adsorption at 0 K. Within the first model system, two different fragment-based clusters were considered where the structure of the fragments was taken from the crystal structure [2]. The model frag1 has one metal cluster and six organic linkers (i.e., Zn_4_O(CO_2_Ph)_6_) as shown in Figure 2A and was used to optimize the H_2_ configuration at the α, β and γ sites. The model frag2 has one phenylene group and two metal clusters as shown in Figure 2B and was used to optimize the H_2_ configuration at the δ and ε sites. The M06-2X and ωB97XD functionals were used in conjunction with the LANL2DZ basis set on all atoms. Subsequent single point calculations using the cc-pVDZ-PP [26,27] basis set then determined the stabilization energy (SE) which represents the interaction strength between hydrogen and IRMOF-1, defined as

(1) SE = E(H_2_ + IRMOF-1) − E(H_2_) − E(IRMOF-1)

In the calculation of SE, the basis set superposition error (BSSE) has been corrected by the counterpoise (CP) method [28,29]. For the sake of simplicity, the notation “method/basis set” (e.g., M06-2X/LANL2DZ) is used to define each calculation.

A second, cell based, cluster model system was constructed wherein the hydrogen configurations in the cells were optimized using the same method and basis set. In this case, a single cubic super-cell cluster is constructed that consists of a face-cube cell and an edge-cube cell, whose coordinates were taken from the crystal structure (Figure 1) [30]. There are 508 atoms in this super-cell cluster, with the α and δ sites being present in the face-cube and the β, γ and ε sites present in the edge-cube. Through the rotation of hydrogen on these sites, different H_2_ configurations were optimized on each primary site. The interaction between adsorbed hydrogen molecules was then investigated upon population of all sorption sites simultaneously. There are eight α sites, eight β sites and twenty-four γ sites around the twelve metal clusters in a super-cell, along with twelve δ sites and twelve ε sites around the 20 phenylene groups. When all possible sites in a super-cell are populated, there are a total 64 adsorbed H_2_ molecules. The interaction among these H_2_ molecules has been investigated using the following approach. A single H_2_ adsorbed at each site was optimized in the super-cell cluster, then all 64 optimized configurations were combined to create the initial guess geometry for the fully H_2_ saturated super-cell. The adsorption geometries of the 64 hydrogen molecules were then re-optimized. The SE of each adsorbed H_2_ molecule at the maximal loading is calculated as

(2) SE = E(IRMOF-1(64 H_2_ adsorbed)) − E(H_2_) − E(IRMOF-1(63 H_2_ adsorbed))

The difference of the SE values for each site before and after simultaneous optimization is used to represent the change of the stabilization energy as a result of maximal loading. The interaction between hydrogen molecules was further investigated by NBO analysis [25]. All the calculations including NBO analyses were performed in GAMESS (M.S. Gordon, Amsterdam, The Netherlands) [31] and the rest in Gaussian09 (Gaussian, Inc., Wallingford, VT, USA) [32]. 

## 3. Results and Discussion

### 3.1. Benchmarking DFT Methods in Fragment Models

**Figure 3:**
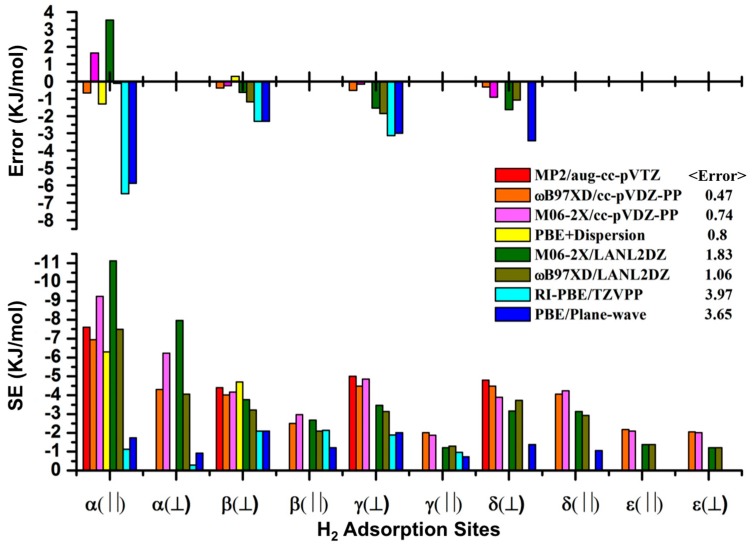
The stabilization energies (SE) of parallel and perpendicular configurations at the five primary sites in frag1 and frag2 model systems. Error is relative to MP2/aug-cc-pVTZ [8].

In addition to several studies that have scanned the potential energy surface (PES) of parallel (||) and perpendicular (⏊) H_2_ configurations [12,13,14], Sillar et al. [9] has optimized the H_2_ configurations at α, β, γ and δ sites using frag1 and frag2 with MP2/def2-TZVP, and the SE values calculated with MP2/aug-cc-pVTZ (Table S2). These data are close to the initial heat of H_2_ sorption −7 kJ/mol [33,34] and the range of adsorption enthalpies of H_2_ between −3.5 and −7.4 kJ/mol depending on whether the site is near the metal node or the organic linker [17]. As such, the SE values based upon MP2 optimization of the various H_2_ configurations in frag1 and frag2 have been chosen here as the reference values for determining the ability of different density functionals to describe the weak dispersive interaction between H_2_ and IRMOF-1. Prior DFT studies have investigated the SE values of different sorption sites using frag1 and frag2 (Table S2), however only PBE and PBE with dispersion corrections have been used [9,13]. As anticipated, there are clear distinctions between the SE values and order of the energetic stability of the sorption sites when dispersion is not accounted for, and the dispersion correction to PBE was required in order for even qualitative agreement with MP2. In this work, the H_2_ configurations in frag1 and frag2 were first optimized and the SE values were compared using the LANL2DZ and then the cc-pVDZ-PP basis sets (Table S2) using M06-2X and ωB97XD, with a detailed comparison to the prior MP2 and PBE + dispersion studies. Figure 3 graphically represents the errors of the different density functionals and basis sets relative to the benchmark MP2 data for sorption to frag1 and frag2.

Both M06-2X and ωB97XD with LANL2DZ basis set represent significant improvements to the PBE data, being very close to the MP2 results, and having average errors relative to MP2 of 1.05 kJ/mol and 1.83 kJ/mol, respectively for ωB97XD and M06-2X functionals. The SE values using M06-2X/LANL2DZ are 11.13 kJ/mol, 3.77 kJ/mol, 3.47 kJ/mol, and 3.17 kJ/mol at the α, β, γ and δ sites, respectively. This represents the same order of stability as experiment (α >> β > γ > δ ≈ ε) [10]. Increasing the basis set size to cc-pVDZ-PP decreases the errors of the SE values relative to MP2 to 0.47 kJ/mol and 0.74 kJ/mol respectively for ωB97XD and M06-2X functionals. The SE values using ωB97XD/cc-pVDZ-PP provide the best overall relation to the MP2 data, with the largest difference between ωB97XD/cc-pVDZ-PP and MP2 being only 0.66 kJ/mol.

The largest impact observed when using the cc-pVDZ-PP basis set comes from the relative energy of the α site—leading to the prediction that the most stable configuration is parallel rather than perpendicular. The other SE values on the β, γ, δ and ε sites do exhibit small changes when the basis set is increased from LANL2DZ to cc-pVDZ-PP. While they are of a small magnitude, they do change the order of energetic stability. Specifically, ωB97XD/LANL2DZ predicts that the δ(||) to be more stable than the δ(⏊), however increasing the basis to cc-pVDZ-PP reverses this trend. High level MP2 calculations of the parallel and perpendicular adsorbed configurations of H_2_ on benzene also indicate that the perpendicular configuration is the favored geometry [12,18,35]. Comparing the optimized geometries between DFT and MP2 indicates that ωB97XD provides the most similar geometries (Table S3).

Compared to the MP2 results, both M06-2X and ωB97XD using the cc-pVDZ-PP basis set exhibit somewhat improved performance relative to the prior PBE + dispersion calculations. These data further support the fact that it is important to use DFT that accounts for dispersion interactions either implicitly or through corrections when studying the interaction between hydrogen and IRMOF-1 (both in terms of the energetics and geometrical parameters). The most accurate stabilization energies of the adsorbed hydrogen have been determined using ωB97XD/cc-pVDZ-PP at the optimized configuration from ωB97XD/LANL2DZ. The M06-2X/LANL2DZ method is a good approach to search H_2_ configurations and predict their interaction strengths at an improved computational cost relative to ωB97XD, however it has slightly poorer performance.

### 3.2. DFT Optimization of H_2_ Configurations in the Super-Cell Cluster as a Function of Concentration

The individual configurations of H_2_ adsorbed to each of the surface sites were first optimized within the IRMOF-1 super-cell cluster. This model system allows for the comparison of H_2_ stabilization energies in the extended crystal framework relative to the fragment based models, as well as the study of perturbations in the stabilization energies that may occur due to site-to-site interactions between H_2_ molecules at higher loading. The twelve optimized H_2_ configurations in the super-cell are illustrated in Supplementary Materials along with their coordinates. Using M06-2X/LANL2DZ and ωB97XD/LANL2DZ, four minima on the parallel configuration potential energy surface were optimized by rotating H_2_ on the α site, α(||)_1–4_, while only one perpendicular configuration was optimized α(⏊). Five hydrogen configurations were also optimized on the β site, one parallel, β(||), which is predicted to have the smallest stabilization energy among the β site configurations, while the others, β(⏊)_1–4_, are the minima on the perpendicular configuration potential energy surface. Two configurations were optimized at the γ site. Note that a third configuration has been previously reported at the γ site, termed III(B) in reference [14], however it was not found to be a stable structure. This is not surprising, given that the SE for this configuration was reported to only be 0.73 kJ/mol using PBE without dispersion corrections. All of these optimized hydrogen configurations on α, β and γ sites are shown in Supplementary Materials (Figure S1). Eight optimized H_2_ configurations on the δ and ε sites were optimized and these optimized H_2_ configurations are shown in Supplementary Materials (Figure S2). The M06-2X functional predicts that the most stable configuration on the δ site is the perpendicular δ(⏊). The other configurations on the δ site are the parallel δ(||)_1–4_ wherein the hydrogen molecule lies on a plane that is parallel to the phenylene plane. The most stable parallel hydrogen, δ(||)_1_, lies over the y axis (coordinate axes shown in Figure 2). When hydrogen rotates from lying along the *y*-axis to *x*-axis, another three parallel configurations are obtained. The stabilization energies of these configurations decrease as the H_2_ is rotated. Hydrogen has three configurations when it is close to the edge of phenylene group on the ε site. The most stable configuration is the ε(⏊)_1_, in which the hydrogen molecule is perpendicular to the phenylene plane. The next stable configuration is the ε(||) in which the hydrogen is on the phenylene plane, and parallel to a phenylene edge. The third configuration is the ε(⏊)_2_, in which the hydrogen is on the phenylene plane, but perpendicular to a phenylene edge.

The stabilization energies of all configurations in the super-cell are listed in Table 1. It is obvious that the PBE calculation without the dispersion correction is very different from M06-2X and ωB97XD, while BSSE corrections also have an important impact upon the energy values. SE values of twenty optimized H_2_ in IRMOF-1 super-cell, model fragments with and without BSSE corrections are listed in Supplementary Materials (Table S4). Within the current data, the BSSE correction in M06-2X/LANL2DZ reduces the SE values by 2.1–4.4 kJ/mol at the α, β and γ sites, and reduces the SE values by 0.7–1.8 kJ/mol at the δ and ε sites. A similar range of corrections is observed using ωB97XD/LAN2DZ. When comparing the SE values between model frag1 and the super-cell (Table 1), the absolute values and the energy order of configurations of the α, β and γ sites are very similar. However, the same cannot be said for the δ sites when comparing frag2 data to that obtained from the super-cell. Using M06-2X within the super-cell, the SE value of ε(⏊)_1_ is 0.04 kJ/mol lower than that of ε(||), however their energy order reverses in model frag2, such that ε(⏊)_1_ has a 0.17 kJ/mol higher SE value than ε(||). Similar behavior is observed using ωB97XD. The SE values of δ(⏊) and δ(||)_1_ also change significantly when using a super-cell. In the M06-2X approach, the SE difference between δ(⏊) and δ(||)_1_ configurations increases from 0.03 to 0.09 kJ/mol when the investigated system changes from the model frag2 to the super-cell. These data provide an initial assessment of the energetic impact caused by long-range affects induced within the crystal lattice relative to the model fragments, a feature that has only been previously inferred by comparing the stabilization energies of different sites as a function of the size of different fragment models [11].

**Figure 4:**
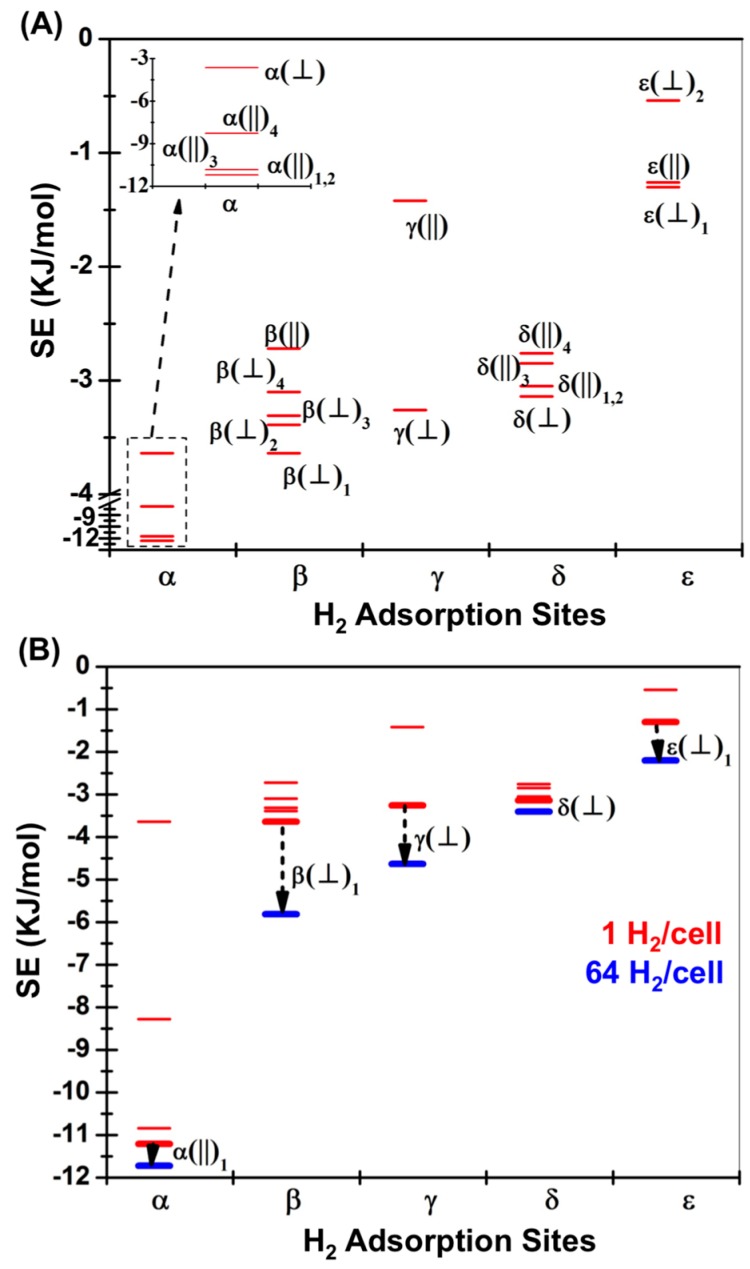
The stabilization energies of H_2_ configurations in the super-cell using M06-2X/LANL2DZ. (**A**) The energy distribution of twenty configurations; (**B**) The change in SE values of the most stable site type with the occupation of 64 H_2_ in the super-cell.

**Figure 5:**
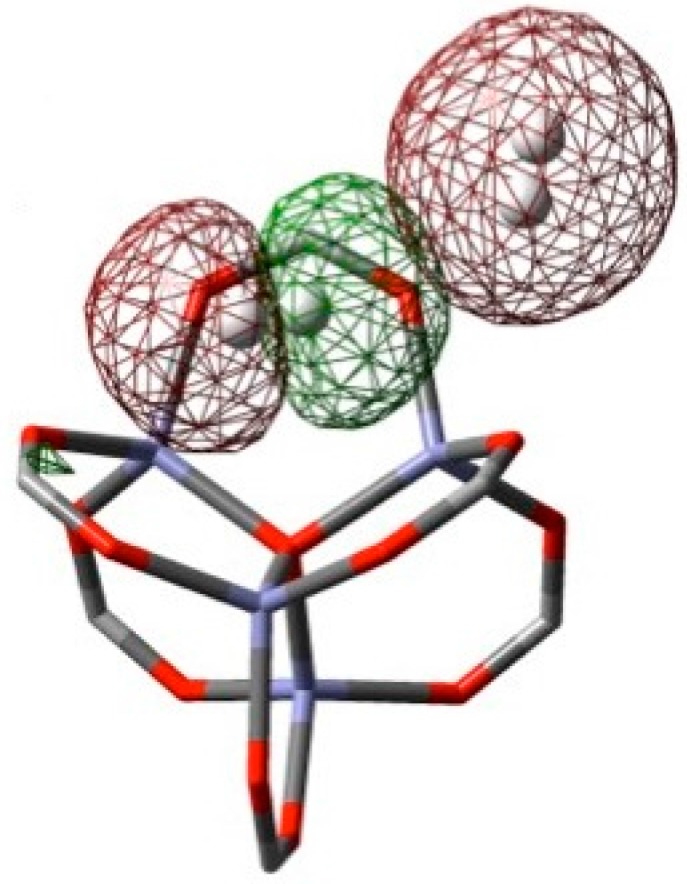
The interaction between hydrogen molecules according to NBO analysis, σ(H_γ_–H_γ_) donation into → σ*(H_α_–H_α_).

The effect of intermolecular interactions between sorbate molecules has been investigated by populating all the primary sites with adsorbed H_2_. The initial 64 hydrogen molecules were placed in the super-cell cluster at the most stable H_2_ configuration obtained from optimization of each individual site in isolation. All 64 positions were then simultaneously optimized using M06-2X/LANL2DZ. The coordinates of super-cell IRMOF-1 and the coordinates of 64 H_2_ adsorbed at optimized positions are listed in Supplementary Materials (Tables S5 and S6, respectively). A new set of SE values of H_2_ on each site was then calculated after the optimization of highly H_2_ loaded system. The new SE values are listed in Table 1 (64 H_2_/cell) and Figure 4 plots the SE values of different sites obtained by optimization of individual H_2_ configurations (1 H_2_/cell) and with all positions filled (64 H_2_/cell). The SE values of the γ site is stabilized by 1.4 kJ/mol when in the presence of the other H_2_ molecules. The change of SE value indicates non-negligible intermolecular interactions among these hydrogen molecules, a feature explored by NBO analysis [25]. All possible interaction energies between bond Natural Bond Orbitals (NBOs) and anti-bond NBOs have been estimated by MP2. The result is a predicted electron donation from bonding NBOs to anti-bonding NBOs (charge transfer). Specifically, the σ(H_γ_ + H_γ_) bonding orbital of the γ-bound H_2_ donates into the anti-bonding orbital of α–bound hydrogen, σ*(H_α_–H_α_). Here the H_γ_ and H_α_ are the 1s atomic orbitals of the γ– and α–bound hydrogen molecules, respectively. Representative pictures of the natural bonding and anti-bonding orbitals are plotted in Figure 5. The interaction energy between these neighboring H_2_ is ~0.79 kJ/mol. The optimized distance between donor hydrogen and acceptor hydrogen atoms is 3.21 Å.

**Figure 6:**
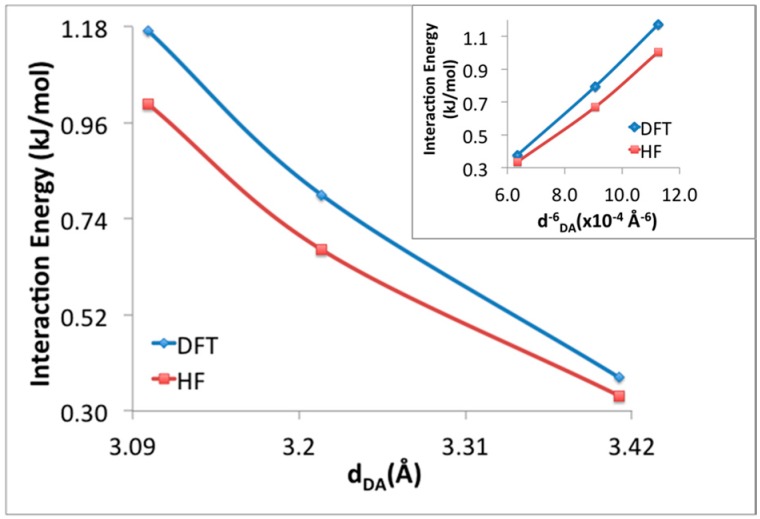
The interaction energies between γ–H_2_ and α–H_2_ molecules with respect to the distance between donor and acceptor H_2_ molecules.

**Figure 7:**
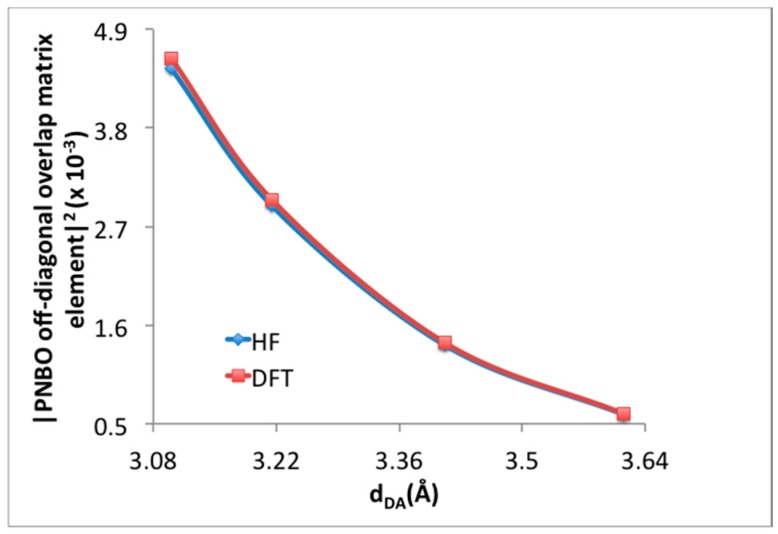
Square of the PNBO off-diagonal overlap matrix element with respect to the distance between donor and acceptor between γ–H_2_ and α–H_2_ molecules.

NBO is not traditionally employed to study weak intermolecular interactions, and as such it may be possible that the charge transfer is merely a manifestation of enhanced dispersion interactions that should occur at high loading. In this work, NBO analysis is preferred to study charge transfer over Symmetry-Adapted Perturbation Theory (SAPT) because charge transfer is enclosed partially in other energy terms of SAPT instead of being explicitly stated [36]. To further study the source of this interaction, the MP2 interaction energies between donor and acceptor H_2_ molecules at the γ and α positions were studied along the potential energy surface where the donor hydrogen molecule was moved toward the center of the super-cell and all other atoms and H_2_ were kept fixed. The PES scan began at a distance of 3.98 Å and ended at 3.58 Å. The interaction energies were first calculated with M06-2X/LANL2DZ, and then using HF with LANL2DZ as a comparison. Thus, the MP2 orbital interaction energies are examined using the NBOs from two methods, one capable of describing dispersion and charge transfer (M06-2X), and one that cannot capture the essential physics of dispersion, but does describe charge-transfer rather well (HF). The interaction energies between donor and acceptor H_2_ molecules with respect to the distance between these H_2_ molecules are given in Figure 6. The perturbation to convert unperturbed NBOs to final Natural Localized Molecular Orbitals (NLMOs) in order to obtain the second-order energies are provided by off-diagonal element, F_i,j_ (F_i,j_ = <Ω_i_|F|Ω*_j_>). These second-order energies are generally proportional to the square of the orbital overlap. As the NBOs are orthogonal, the overlap of the pre-orthogonal NBOs (which neglect NBO’s interatomic orthogonalization step) has also been examined, and in presented in Figure 7. Although HF level of theory cannot capture dispersion, the interaction energies and the square of the off-diagonal NBO Fock matrix elements obtained after HF calculation are very close to those obtained after M06-2X calculations and show the same pattern with respect to distance. This provides evidence that the neighboring γ–H_2_, and α–H_2_ participate in site-to-site charge transfer interactions that significantly stabilize H_2_ adsorption. Importantly, we confirm that this interaction does not only exist at the theoretical maximum loading of 64 adsorbed H_2_ within the super cell. Indeed, merely calculating two isolate H_2_ molecules at the geometry fixed within the super-cell using NBO leads to an interaction energy of ~0.46 kJ/mol.

## 4. Conclusions

Twenty hydrogen configurations on five primary sites were optimized using M06-2X/LANL2DZ in a super-cell consisting both of an edge and face cube sub-cells of IRMOF-1 (MOF-5). Their stabilization energy order is α(||)_1_ >> β(⏊)_1_ > γ(⏊) > δ(⏊) > ε(⏊)_1_, consistent with previous studies. To investigate the possible interaction among adsorbed hydrogen molecules at high pressures, 64 H_2_ molecules sorbed on the five primacy sites were populated and optimized simultaneously. This sorption configuration represents a theoretical maximum with all 20 identified site types populated. Though this configuration (14 wt %) has not been achieved experimentally (11.5 wt %), it does represent a suitable model system to explore neighboring adsorbate interactions. The high hydrogen concentration significantly stabilizes the γ site. Natural bond order analysis reveals that delocalization of electrons, or charge transfer, from the σ orbitals of the H_2_ bound at the γ positions into the σ* orbitals of H_2_ bound at the α sites leads to stabilization of the interaction of H_2_ at the γ by 1.4 kJ/mol, respectively (using M06-2X/LANL2DZ). Indeed, independent NBO calculation of only the two adsorbed α- and γ-bound H_2_ at the same geometries as in in the super cell yields similar observations. Given that the distance between these neighboring H_2_ molecules is only 3.21 Å, their site-to-site interactions should not be ignored in future computational studies at modest to high H_2_ pressures, and point to a general consideration of loading when examining the adsorption of weakly bound small molecules to porous media.

## Figures and Tables

**Table 1:** Stabilization energies (kJ/mol) of twenty H_2_ configurations in the super-cell. The values in parentheses are based on calculations using frag1 and frag2.

H_2_ Position	PBE in VASP [14]		M06-2X (1 H_2_/Cell)	M06-2X (64 H_2_/Cell)	ΩB97XD (1 H_2_/Cell)
α(||)_1_	II(B)	−1.31	−11.21 (−11.13)	−11.72	−9.37 (−9.24)
α(||)_2_	II(C)	1.73	−11.21	–	−9.41
α(||)_3_	II(D)	−1.28	−10.84	–	−9.41
α(||)_4_	II(E)	−1.73	−3.64	–	−9.41
α(⏊)	II(A)	−0.92	−8.28 (−7.96)	–	−6.61 (−6.23)
β(⏊)_1_	I(A)	−2.09	−3.64 (−3.77)	−5.81	−4.05 (−4.16)
β(⏊)_2_	I(B)	−1.92	−3.39	–	−4.01
β(⏊)_3_	I(D)	−1.95	−3.31	–	−3.81
β(⏊)_4_	I(E)	−1.43	−3.10	–	−4.02
β(||)	I(C)	−1.21	−2.72 (−2.68)	–	−3.14 (−2.97)
γ(⏊)	III(A)	−2.01	−3.26 (−3.46)	−4.63	−4.85 (−4.85)
γ(||)	III(C)	−1.15	−1.42 (−1.21)	–	−2.22 (−1.87)
γ	III(B)	−0.73	–	–	–
δ(⏊)	IV(A)	−1.38	−3.14 (−3.17)	−3.40	−4.06 (−3.89)
δ(||)_1_	IV(B)	−1.06	−3.05 (−3.14)	–	−4.35 (−4.23)
δ(||)_2_	IV(C)	−1.02	−3.05	–	−4.27
δ(||)_3_	IV(D)	−0.96	−2.85	–	−2.97
δ(||)_4_	IV(E)	−0.92	−2.76	–	−4.06
ε(⏊)_1_	V(A)	−1.22	−1.30 (−1.21)	−2.20	−2.30 (−2.01)
ε(||)	V(B)	−0.98	−1.26 (−1.38)	–	−2.22 (−2.09)
ε(⏊)_2_	V(C)	−0.50	−0.54	–	−1.21

## Supplementary Materials

The supplementary materials are available online at www.mdpi.com/1996-1944/9/7/578/s1.

## Abbreviations

The following abbreviations are used in this manuscript:

IRMOFs	Isoreticular metal organic frameworks
QM	Quantum Mechanical
NBO	Natural Bond Order
MP2	Møller-Plesset 2nd order perturbation theory
BDC	1,4-benzenedicarboxylate
BSSE	basis set superposition error
DFT	Density Functional Theory
EDA	Energy Decomposition Analysis
NBO	Natural Bond Order
HF	Hartree-Fock
CP	counterpoise
PES	Potential Energy Surface
NBOs	Natural Bond Orbitals
SE	Stabilization Energy
NLMOs	Natural Localized Molecular Orbitals
SAPT	Symmetry-Adapted Perturbation Theory
