# Supplementary material for: H2 Adsorbed Site-to-Site Electronic Delocalization within IRMOF-1: Understanding Non-Negligible Interactions at High Pressure
Source: Materials (Basel). 2016 Jul 15;9(7):578. doi: 10.3390/ma9070578 (PMC5456847; doi:10.3390/ma9070578)
Supplement: Supplementary file 1 [file materials-09-00578-s001.pdf]

Supplementary Materials: H_2 Adsorbed Site-to-Site Electronic Delocalization Within IRMOF-1: Understanding Non-negligible Interactions at High Pressure

Jian Wu,* Mustafa Kucukkal, Aurora E. Clark*

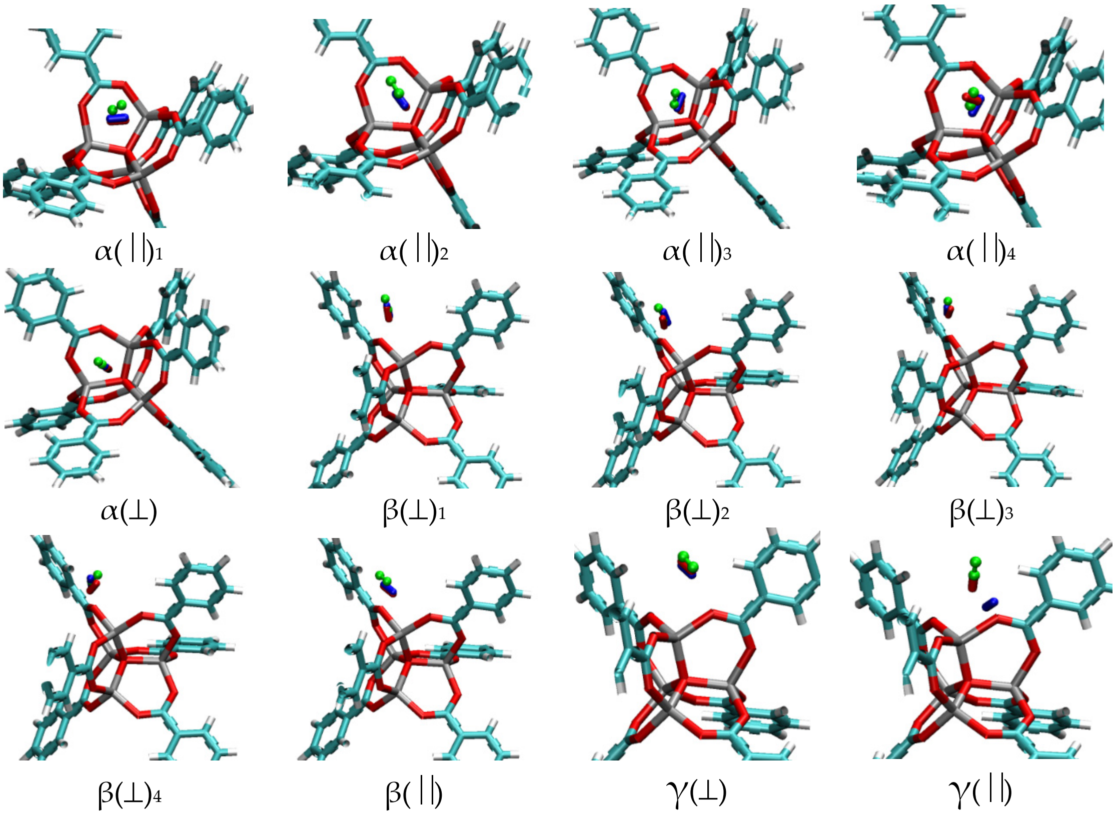

Figure S1. Twelve optimized hydrogen configurations on α, β and γ sites.

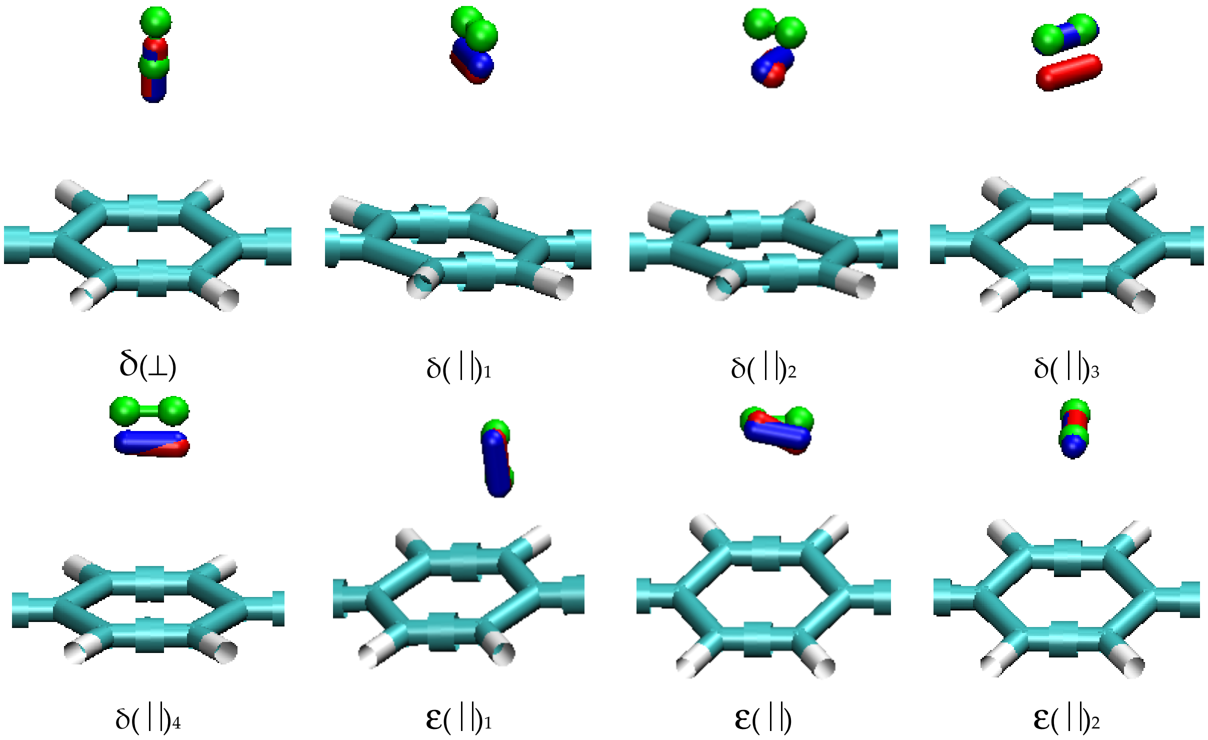

Figure S2. Eight optimized hydrogen configurations on δ and ε sites around phenyl group.

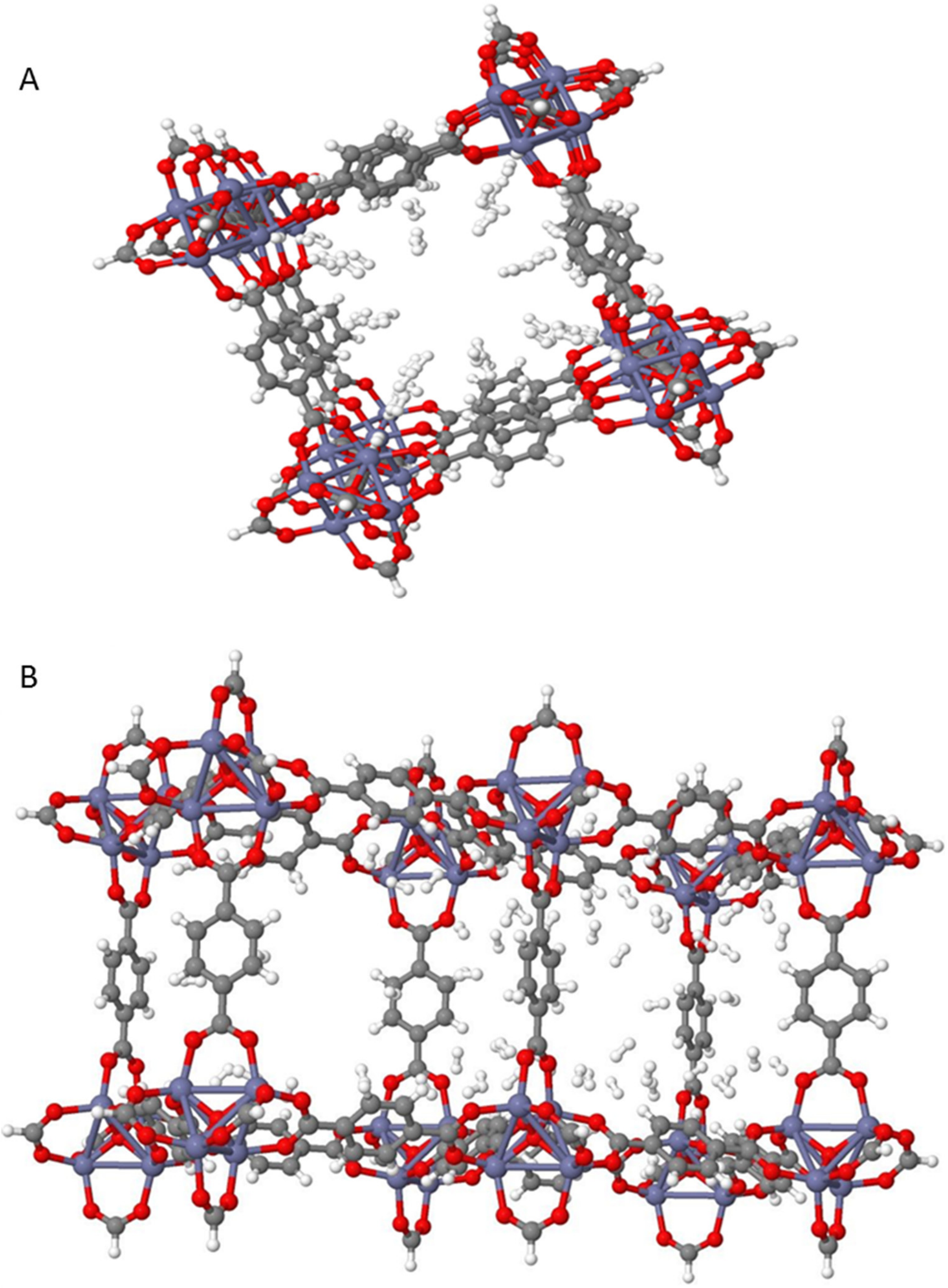

Figure S3. Representation of super-cell cluster IRMOF-1 with optimized 64 H_2 molecules. (**A**) Front view; (**B**) side view.

Table S1. Compilation of prior and current quantum mechanical studies (excluding AIMD), including the chemical models, methods, basis sets, and type of calculation used to examine H_2 sorption to IRMOF-1.

Fragment Chemical Models						
**Fragment**	**Method**	**Basis**	**Type**	**Cbs**	**Bsse**	**Ref**
H_2-BDC-H_2	MP2<br>–<br>–<br>–<br>B3LYP<br>HF	aug-cc-pVDZ<br>aug-cc-pVTZ<br>aug-cc-pVQZ<br>cc-PVTZ<br>6-31g(d)<br>cc-pVTZ	Opt<br>SP<br>SP<br>PES Scan<br>Opt<br>PES Scan	✓	–	11<br>11<br>11<br>13<br>13<br>13
H_2-$OZn_4(CO_2H)_6$	MP2	def2-TZVP<br>aug-cc-pVXZ	Opt<br>SP	✓	✓	8
H_2-$OZn_4(CO_2Ph)_6$	MP2	def2-TZVP<br>aug-cc-pVXZ	Opt<br>SP	✓	✓	8
H_2-$Zn_4O(HCO_2)_6$	MP2<br>–<br>RI-MP2<br>B3LYP<br>HF<br>MP2	SVP/cc-pVDZ<br>TZVP/aug-cc-pVTZ<br>TZVPP<br>631g(d)<br>cc-pVTZ<br>LANL2DZ/aug-cc-pVQZ	Opt<br>SP<br>PES Scan<br>Opt<br>PES Scan<br>PES Scan	✓	✓	11<br>11<br>12<br>13<br>13<br>16
(Zn4O)(HCO2)5·BDC·Li	RI-MP2<br>CCSD(T)	TZVPP<br>TZVPP	opt<br>opt	–	–	17<br>17
**Unit Cell Chemical Models**						
**# Atoms/Cell**	**Method**	**Basis**	**Type**	**Cbs**	**Bsse**	**Ref**
108	LDA	plane-wave	H_2 opt/cell fixed	–	–	4
NA	PBE	6-31G*	cell opt	–	–	11
358	RI-DFT PBE	TZVPP	H_2 PES Scan	–	✓	12
NA	BLYP	DN	SP	–	–	16
106	GGA	plane-wave	H_2 opt/cell opt	–	–	18
106	LDA	plane-wave	H_2 opt/cell opt	–	–	19
64	PBE<br>PBE+Disp	plane-wave<br>plane-wave	H_2 opt/cell-opt<br>H_2 opt/cell-opt	–	–	8<br>8

Table S2. Stabilization energies (kJ/mol) of an adsorbed hydrogen molecule in frag1 which consists of one metal cluster and six organic linkers ($Zn_4O(CO2Ph)_6$), and frag2 which consists of one phenylene group and two metal clusters ($Ph(CO2)(Zn_4O)_2$). The different sorption sites are denoted for parallel (||) and perpendicular (⊥) H_2 configurations.

Method	Frag1						Frag2			
	α(||)	α(⊥)	β(⊥)	β(||)	γ(⊥)	γ(||)	δ(⊥)	δ(||)	ε(||)	ε(⊥)
MP2 [8] [a]	−7.6	–	−4.4	–	−5.0	–	−4.8	–	–	–
RIMP2 [12] [b]	−3.10	−1.51	−1.05	−1.34	−1.80	−0.54	–	–	–	–
PBE [14] [c]	−1.73	−0.92	−2.09	−1.21	−2.01	−0.73	−1.38	−1.06	−0.50	−0.98
RI-PBE [12] [d]	−1.13	−0.29	−2.09	−2.13	−1.88	−0.96	–	–	–	–
PBE + Dis[8] [e]	−6.30	–	−4.70	–	–	–	–	–	–	–
M06-2X/ LANL2DZ	−11.13	−7.96	−3.77	−2.68	−3.46	−1.21	−3.17	−3.14	−1.38	−1.21
ωB97XD/ LANL2DZ	−9.24	−6.23	−4.16	−2.97	−4.85	−1.87	−3.89	−4.23	−2.09	−2.01
M06-2X/ cc-pVDZ-PP	−7.49	−4.06	−3.22	−2.09	−3.14	−1.30	−3.72	−2.93	−1.38	−1.21
ωB97XD/cc-pVDZ-PP	−6.94	−4.31	−4.02	−2.51	−4.48	−2.01	−4.48	−4.06	−2.18	−2.05

[a] MP2 with BSSE correction and optimization of H_2; [b] RIMP2 with BSSE; [c] PBE without BSSE; [d] RI-PBE with BSSE; [e] PBE with dispersion and BSSE.

Table S3. Relevant geometric parameters (in Å) of adsorbed H_2 in frag1 and frag2. Distances are with respect to the center of H_2 for the parallel orientation, and the closest hydrogen atom for the perpendicular orientation.

H_2 Congfiurations at Sorption Sites	MP2[1]		RI-MP2[2]	RI-PBE[2]	M06-2X		ωB97XD	
	$d_{H–H}$	$d_{H–MOF}$	$d_{H–MOF}$	$d_{H–MOF}$	$d_{H–H}$	$d_{H–MOF}$	$d_{H–H}$	$d_{H–MOF}$
α(‖)	0.741	2.92 [a]	4.0 [a]	3.9 [a]	0.74	3.20 [a]	0.76	3.40 [a]
α(⊥)	–	–	4.0 [a]	3.6 [a]	0.74	2.89 [a]	0.74	3.17 [a]
β(⊥)	0.739	2.99[a]	3.8 [c]	3.5 [c]	0.74	2.89 [a] (3.14 [c])	0.74	2.82 [a] (3.22 [c])
β(‖)	–	–	3.4 [c]	3.5 [c]	0.74	2.99 [a] (3.17 [c])	0.74	3.07 [a] (3.28 [c])
γ(⊥)	0.739	2.97[a]	3.8 [c]	3.8 [c]	0.74	2.84 [a] (3.47 [c])	0.74	2.75 [a] (3.43 [c])
γ(‖)	–	–	4.2[c]	4.2 [c]	0.74	3.55 [a] (4.27 [c])	0.74	3.38 [a] (4.08 [c])
δ(⊥)	0.739	3.35[b]	–	–	0.74	2.69 [b]	0.74	2.69 [b]
δ(‖)	–	–	–	–	0.74	2.90 [b]	0.74	2.96 [b]
ε(‖)	–	–	–	–	0.74	3.56 [d]	0.74	3.40 [d]
ε(⊥)	–	–	–	–	0.74	3.42 [d]	0.74	3.33 [d]

[a] Distance (in Å) to the closest oxygen atom (which is the tetrahedrally O atom for the α sites); [b] Distance (in Å) to the center of benzene ring; [c] Distance (in Å) to the nearest Zn atom; [d] Distance (in Å) to the nearest C atom of benzene ring.

Table S4. SE values of twenty optimized H_2 in IRMOF-1 super-cell, model fragments (in parentheses). The SE values without BSSE correction are also listed. The green ball-bond models are the PBE optimized configurations. The blue tube models are the M06-2X/LANL2DZ optimized configurations. The red tube models represent the ωB97XD/LANL2DZ optimized configurations.

Representation of H_2 and Super-cell at Sorption Sites	H_2 Configurations at Sorption Sites	M06-2X			ωB97XD		
		Coordinate	BSSE	No BSSE	Coordinate	BSSE	No BSSE
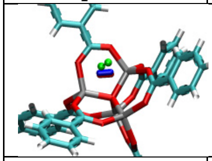	α(‖)1	(2.00,1.43,−2.04)<br>(1.75,2.06,−1.72)	−11.21<br>(−11.13)	−15.6	(2.14,1.55,−2.15)<br>(1.90,2.17,−1.82)	−9.41	−12.97
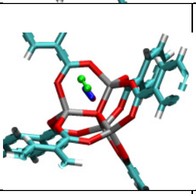	α(‖)2	(1.75,2.06,−1.72)<br>(1.99,1.43,−2.04)	−11.21	−15.6	(1.88,2.15,−1.82)<br>(2.16,1.55,−2.16)	−9.37<br>(−9.24)	−12.93
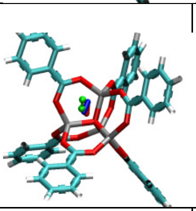	α(‖)3	(1.72,1.96,−1.58)<br>(2.07,1.56,−2.10)	−10.84	−15.2	(1.81,2.13,−1.85)<br>(2.19,1.57,−2.16)	−9.41	−12.93
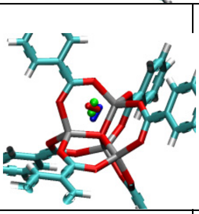	α(⊥)	(2.13,2.13,−2.02)<br>(1.66,1.66,−1.69)	−8.28<br>(−7.96)	−12.7	(2.24,2.24,−2.19)<br>(1.76,1.76,−1.88)	−6.61<br>(−6.23)	−10.04
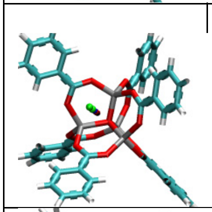	α(‖)4	(2.61,2.35,−2.35)<br>(2.61,2.88,−2.88)	−3.64	−5.77	(1.85,2.15,−1.83)<br>(2.17,1.56,−2.15)	−9.41	−12.97
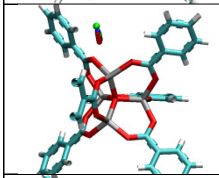	β(⊥)1	(2.86,2.86,3.06)<br>(3.33,3.33,3.39)	−3.64<br>(−3.77)	−6.74	(2.97,2.97,2.98)<br>(3.40,3.403.41)	−4.05<br>(−4.16)	−6.61
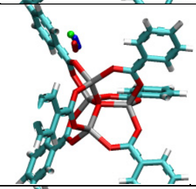	β(⊥)2	(2.79,2.92,3.03)<br>(3.29,3.15,3.52)	−3.39	−6.57	(3.05,2.95,2.93)<br>(3.45,3.48,3.27)	−4.01	−6.57
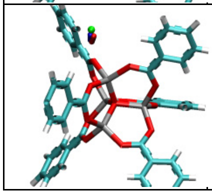	β(⊥)3	(2.61,3.24,2.74)<br>(3.21,3.22,3.17)	−3.31	−6.61	(2.59,3.39,2.74)<br>(3.11,3.43,3.27)	−3.81	−6.57

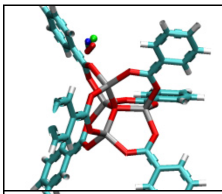	β(⊥)$_4$	(3.22,3.16,3.11)<br>(2.51,3.19,2.89)	−3.10	−6.40	(3.19,3.56,3.24)<br>(2.68,3.37,2.74)	−4.02	−6.69
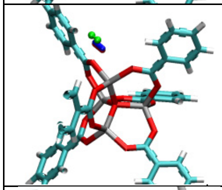	β(∣∣)	(2.84,3.24,2.94)<br>(3.09,2.55,3.03)	−2.72<br>(−2.68)	−6.02	(2.94,3.30,2.98)<br>(3.18,2.60,3.08)	−3.14<br>(−2.97)	−5.90
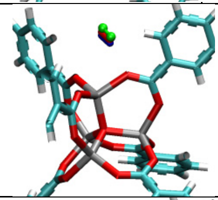	γ(⊥)	(3.57,3.57,1.16)<br>(4.09,4.09,1.27)	−3.26<br>(−3.46)	−6.19	(3.42,3.42,0.06)<br>(3.89,3.89,−0.30)	−4.85<br>(−4.85)	−7.24
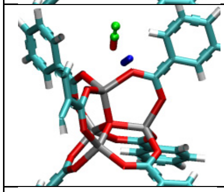	γ(∣∣)	(4.17,4.17,0.97)<br>(4.09,4.09,1.70)	−1.42<br>(−1.21)	−3.64	(4.04,4.04,0.92)<br>(3.96,3.96,1.65)	−2.22<br>(−1.87)	−4.39
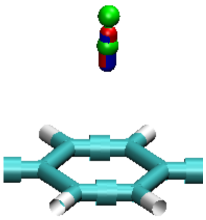	δ(⊥)	(1.91,6.42,−1.90)<br>(2.43,6.42,−2.43)	−3.14<br>(−3.17)	−4.52	(1.90,6.42,−1.90)<br>(2.43,6.42,−2.42)	−4.06<br>(−3.89)	−5.36
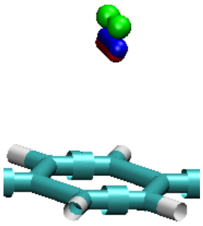	δ(∣∣)$_1$	(2.32,6.42,−1.78)<br>(1.79,6.41,−2.30)	−3.05<br>(−3.14)	−4.81	(2.37,6.42,−1.92)<br>(1.82,6.43,−2.43)	−4.35<br>(−4.23)	−5.77
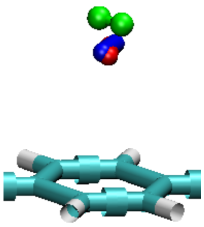	δ(∣∣)$_2$	(1.80,6.33,−2.30)<br>(2.31,6.52,−1.79)	−3.05	−4.81	(1.88,6.21,−2.31)<br>(2.31,6.63,−1.88)	−4.27	−5.77
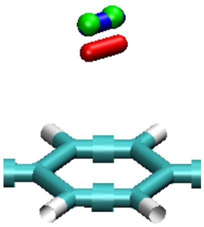	δ(∣∣)$_3$	(1.91,6.11,−2.21)<br>(2.21,6.72,−1.91)	−2.85	−4.64	(2.33,6.07,−2.63)<br>(2.65,6.67,−2.34)	−2.97	−3.93

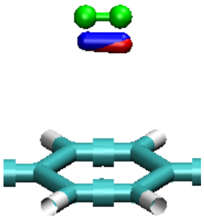 δ(││)4	(2.11,6.10,−2.05)<br>(2.04,6.84,−2.08)	−2.76	−4.56	(2.10,6.06,−2.10)<br>(2.09,6.81,−2.09)	−4.06	−5.65
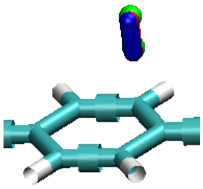 ε(⊥)1	(3.57,6.45,3.04)<br>(3.05,6.45,3.56)	−1.30<br>(−1.21)	−2.51	(3.54,6.45,3.01)<br>(3.01,6.47,3.53)	−2.30<br>(−2.01)	−3.43
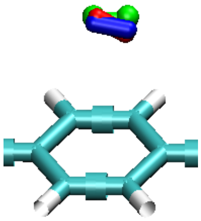 ε(││)	(3.54,6.14,3.13)<br>(3.25,6.75,3.42)	−1.26<br>(−1.38)	−2.43	(3.32,6.08,3.18)<br>(3.21,6.81,3.30)	−2.22<br>(−2.09)	−3.35
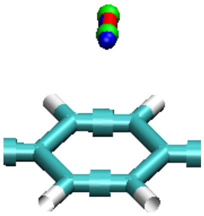 ε(⊥)2	(3.84,6.41,3.84)<br>(3.32,6.41,3.32)	−0.54	−1.26	(3.70,6.42,3.70)<br>(3.18,6.42,3.17)	−1.21	−2.01

Table S5. The super−cell geometry of IRMOF−1 (x, y and z coordinates).

Atom	x (Å)	y (Å)	z (Å)
Zn	−13.875891	8.737619	−2.9177
Zn	11.793109	8.736912	−2.919527
O	−12.759174	8.973839	−1.356443
O	12.909827	8.973132	−1.358264
O	−13.575622	10.085119	−4.239579
O	12.093378	10.084411	−4.241404
C	−12.759319	11.098551	−4.238786
C	12.909681	11.097842	−4.240612
Zn	−13.875656	9.210124	0.204982
Zn	11.793348	9.209419	0.203158
O	−15.739258	9.146631	−0.214833
O	9.929742	9.145919	−0.216659
C	−16.339998	8.97394	−1.356183
C	9.329002	8.973236	−1.357975
O	−15.739429	8.801215	−2.497619
O	9.929571	8.800511	−2.499447
O	−11.942967	11.399175	−3.271011
O	13.726032	11.398467	−3.272837
Zn	−11.642543	10.53515	−1.592771
Zn	14.026457	10.534442	−1.594597
Zn	−11.642644	7.412501	−1.120256
Zn	14.026404	7.411753	−1.122096
O	−11.942731	6.548423	0.558109
O	13.726172	6.547753	0.556196
O	−13.575276	7.862604	1.526817
O	12.093717	7.861891	1.524985
O	−13.575221	10.888377	1.068977
O	12.093779	10.887669	1.067151
O	−11.942738	11.857016	−0.245241
O	13.726262	11.856307	−0.247068
O	−9.778981	10.115151	−1.52936
O	15.890019	10.114443	−1.531187
O	−9.779019	7.832355	−1.183949
O	15.889978	7.831659	−1.185767
O	−11.943041	6.090615	−2.46775
O	13.725945	6.089913	−2.46958
C	−12.759402	6.091494	−3.481148
C	12.909585	6.090795	−3.482985
O	−13.575671	7.05935	−3.781739
O	12.093328	7.058641	−3.783565
C	−12.759508	4.919952	−4.344729
C	12.909523	4.919244	−4.346543
C	−11.909815	3.847596	−4.079775
C	13.759131	3.846899	−4.081559
C	−11.909993	2.760685	−4.881081

C	13.759058	2.760072	−4.882702
C	−12.759678	2.696729	−5.983581
C	12.909288	2.696034	−5.985429
C	−12.759905	1.525042	−6.847299
C	12.90928	1.524484	−6.849008
O	−11.943215	0.557561	−6.546394
O	13.725494	0.556634	−6.548412
Zn	−11.643346	−1.120907	−7.410675
Zn	14.025721	−1.12164	−7.412446
Zn	−13.876572	0.204222	−9.208059
Zn	11.792433	0.203517	−9.20989
O	−13.57613	1.526061	−7.860558
O	12.092874	1.525362	−7.862387
C	−13.609307	3.769101	−6.248586
C	12.059688	3.768372	−6.250417
C	−13.609219	4.855923	−5.447441
C	12.059794	4.855209	−5.449272
C	7.873576	8.973258	−1.357974
C	7.167815	9.153053	−0.169842
C	7.167763	8.793534	−2.545807
C	5.817526	9.153105	−0.169773
C	5.817359	8.793586	−2.545745
C	−12.758499	−0.766313	11.856895
C	12.910499	−0.767023	11.855073
C	5.111516	8.973323	−1.357705
O	−11.942197	0.247116	11.857689
O	13.726802	0.246409	11.855866
O	−13.574851	−1.066941	10.889122
O	12.094148	−1.067649	10.887299
C	3.656153	8.97339	−1.357607
Zn	−11.641927	1.594616	10.53581
Zn	14.027072	1.593909	10.533989
Zn	−13.875274	−0.202916	9.210881
Zn	11.79373	−0.203626	9.209062
O	3.055413	8.800697	−2.498957
O	3.055584	9.146112	−0.21617
Zn	1.191812	8.737204	−2.918772
Zn	1.192054	9.209703	0.203905
Zn	−11.643512	−1.593432	−10.533306
Zn	14.025488	−1.594139	−10.535133
Zn	1.191133	0.203803	−9.209129
Zn	−1.041247	10.534858	−1.593525
Zn	1.192431	−0.20333	9.209811
Zn	−13.875957	−8.73631	2.920526
Zn	11.793042	−8.737023	2.9187
Zn	−1.041601	−10.534261	1.594526
Zn	−1.042215	−1.593728	−10.53406

Zn	−1.040631	1.594324	10.53506
Zn	−11.642897	−10.533962	1.59528
Zn	14.026102	−10.534676	1.593453
Zn	1.191745	−8.73673	2.919454
Zn	1.192481	2.91935	8.737308
Zn	−13.875222	2.919765	8.738377
Zn	11.793778	2.919057	8.736554
Zn	−13.876192	−9.208814	−0.202154
Zn	11.792811	−9.209529	−0.203981
Zn	1.19152	−9.209227	−0.20322
Zn	1.191079	−2.918878	−8.736627
Zn	−13.876624	−2.918459	−8.735554
Zn	11.792376	−2.919167	−8.737381
Zn	−1.040809	1.121826	7.41239
Zn	−11.642907	−7.411308	1.122767
Zn	14.026153	−7.411989	1.120952
Zn	−1.041911	−1.121219	−7.41142
Zn	−1.041517	−7.411608	1.122012
Zn	−11.642261	1.122217	7.413281
Zn	14.026837	1.121408	7.411299
Zn	−1.04125	7.412235	−1.120998
O	−12.760008	−1.35716	−8.971877
O	12.909004	−1.357856	−8.973715
O	0.074503	−1.357509	−8.972798
O	0.07534	8.973475	−1.357363
O	0.075838	1.358034	8.97365
O	−12.758646	1.358403	8.974549
O	12.910353	1.357687	8.972727
O	−12.759478	−8.972588	1.359113
O	12.909527	−8.973305	1.357279
O	0.075029	−8.972946	1.358198
O	−11.943898	−0.245914	−11.855143
O	13.725103	−0.246624	−11.856969
O	0.890706	1.067829	−10.88737
O	−0.741016	11.398866	−3.271808
O	0.892196	−1.067338	10.888095
O	−13.575573	−10.083825	4.242362
O	12.093426	−10.084539	4.240538
O	−0.741179	−11.398285	3.272765
O	−15.740148	−0.215675	−9.144382
O	9.928852	−0.216382	−9.146206
O	−0.741995	−3.271999	−11.398098
O	−0.740196	3.272577	11.399054
O	−15.738837	0.217084	9.147471
O	9.930162	0.216375	9.14564
O	−11.943357	−11.855811	0.247793
O	13.725642	−11.856525	0.245966

O	3.055282	−8.800323	2.499374
O	3.056043	2.499351	8.80072
O	−13.574762	4.241615	10.085862
O	12.094237	4.240907	10.084039
O	−15.73973	−9.145219	0.217926
O	9.929269	−9.14593	0.2161
O	0.891074	−10.887487	−1.067221
O	3.054657	−2.498982	−8.800303
O	0.891827	10.887979	1.067948
O	−13.576428	−4.240325	−10.083083
O	12.092572	−4.241033	−10.084911
O	0.891474	−10.084229	4.241335
O	−0.740246	0.246809	11.856896
O	−11.94313	−11.397971	3.273562
O	13.725868	−11.398684	3.271737
O	−13.576341	1.068231	−10.886341
O	12.092658	1.067523	−10.888169
O	−0.741946	−0.246228	−11.855939
O	0.891426	10.08472	−4.240608
O	−0.741406	−11.856127	0.246996
O	3.056004	0.216566	9.146135
O	−11.942146	3.272886	11.399847
O	13.726852	3.272179	11.398026
O	−15.739559	−8.799802	2.500711
O	9.92944	−8.800514	2.498886
O	−11.943947	−3.271684	−11.397301
O	13.725053	−3.272392	−11.399129
O	3.054694	−0.216195	−9.14572
O	−0.740788	11.856708	−0.246038
O	3.055112	−9.145742	0.216589
O	−15.740186	−2.49846	−8.798966
O	9.928814	−2.499168	−8.800796
O	0.89062	−4.240726	−10.084113
O	−15.738799	2.49987	8.802055
O	9.930201	2.499164	8.800237
O	−13.575973	−10.887083	−1.066194
O	12.093026	−10.887797	−1.068018
O	0.892285	4.241217	10.084837
O	−0.7406	2.469318	6.090497
O	0.891091	−7.861744	−1.525096
O	−11.943067	−6.547152	−0.555655
O	13.725721	−6.547968	−0.557293
O	−13.57499	3.783774	7.060093
O	12.094009	3.783067	7.058271
O	0.891756	7.862224	1.525808
O	−0.741606	−2.46874	−6.089539
O	−13.576202	−3.782484	−7.057315

O	12.092798	−3.783192	−7.059142
O	−2.905133	−7.83146	1.185703
O	−9.778453	1.185282	7.832499
O	15.890437	1.184895	7.831124
O	−2.904865	7.832169	−1.184429
O	−9.779745	−1.184528	−7.830707
O	15.88926	−1.185229	−7.832531
O	−2.904233	1.530833	10.115245
O	−2.905753	−1.530135	−10.11398
O	−9.779322	−10.114065	1.531603
O	15.889679	−10.114779	1.529778
O	−11.94354	−2.468427	−6.088741
O	13.725448	−2.469135	−6.090573
O	−0.74105	6.548	0.557356
O	0.890847	−3.782887	−7.058343
O	−0.74143	−6.547558	−0.556348
O	−13.575916	−7.861319	−1.524038
O	12.093063	−7.862014	−1.525858
O	−11.942517	2.469592	6.091249
O	13.72645	2.468921	6.08947
O	0.892061	3.783372	7.059066
O	−2.90558	−1.184715	−7.831192
O	−9.779288	−7.831281	1.186188
O	15.889716	−7.831995	1.184359
O	−2.904409	1.18542	7.832439
O	−9.779911	−1.529945	−10.11349
O	15.889089	−1.530647	−10.115318
O	−2.905164	−10.114261	1.531113
O	−9.77839	1.530957	10.115738
O	15.890609	1.530314	10.113908
O	−2.904824	10.114961	−1.529848
O	−11.943034	−6.089412	2.470303
O	13.725957	−6.090129	2.468478
O	−0.740716	−0.556698	6.548215
O	−0.741707	0.557115	−6.547287
O	0.891376	7.05895	−3.782767
O	0.891526	−7.05846	3.783494
O	−13.575069	−1.524788	7.86336
O	12.093909	−1.525483	7.86152
O	−0.741121	6.090305	−2.468548
O	−11.942365	−0.556259	6.549068
O	13.726403	−0.556855	6.547312
O	−0.741096	−6.089728	2.469507
O	0.891944	−1.525223	7.862346
O	−13.575523	−7.058055	3.784522
O	12.093476	−7.058768	3.782696
O	0.890912	1.525712	−7.861619

C	−12.760144	0.767562	−11.854233
C	12.908856	0.766853	−11.856059
C	0.074356	0.767203	−11.855144
C	0.075181	11.098196	−4.239699
C	0.076	−0.766668	11.855985
C	−12.759326	−11.0973	4.241452
C	12.909673	−11.098014	4.239627
C	0.075173	−11.097659	4.24054
C	−12.758942	11.856188	0.768271
C	12.910058	11.85548	0.766445
C	−16.340822	−1.357052	−8.971631
C	9.328178	−1.357741	−8.97346
C	0.074274	−4.239853	−11.097508
C	0.076084	4.240388	11.098349
C	−16.339473	1.358494	8.974805
C	9.329527	1.357736	8.972987
C	−12.759704	−11.854937	−0.765603
C	12.909296	−11.85565	−0.767428
C	3.655851	−8.973048	1.357944
C	3.656678	1.357943	8.97339
C	−12.758415	4.240742	11.099258
C	12.910584	4.240034	11.097437
C	−16.340299	−8.972495	1.359363
C	9.3287	−8.973204	1.357536
C	0.074796	−11.855296	−0.766515
C	3.655329	−1.357605	−8.973055
C	0.075559	11.855833	0.767358
C	−12.760226	−4.239495	−11.096596
C	12.908775	−4.240203	−11.098422
C	0.07571	3.482758	6.091297
C	0.074941	−6.848256	−1.524068
C	−12.759774	−6.847928	−1.523098
C	12.909427	−6.848598	−1.52506
C	−12.758795	3.483104	6.092201
C	12.910205	3.482398	6.090381
C	−12.759091	6.84916	1.525842
C	12.909979	6.848441	1.524089
C	0.075604	6.848888	1.524882
C	0.074654	−3.482214	−6.090451
C	−12.75985	−3.481859	−6.08954
C	12.909151	−3.482569	−6.091368
C	−3.505795	−8.972854	1.35845
C	−9.177881	1.358542	8.974286
C	16.491178	1.357588	8.972472
C	−3.505498	8.973585	−1.357101
C	−9.179166	−1.357237	−8.972139
C	16.489829	−1.357955	−8.973967

C	−3.504972	1.358138	8.973895
C	−9.178346	8.973742	−1.356691
C	16.490654	8.973033	−1.358522
C	−3.506326	−1.357407	−8.972542
C	−9.178644	−8.972687	1.358848
C	16.490351	−8.973404	1.357025
C	−12.759244	−6.090244	3.483816
C	12.909755	−6.090959	3.481992
C	0.075788	−1.524134	6.849019
C	0.074874	1.524718	−6.848165
C	0.075091	6.091143	−3.482065
C	0.075254	−6.090604	3.482905
C	−12.758995	−1.52386	6.849823
C	12.910127	−1.524667	6.848036
C	7.166872	−0.169653	−9.15307
C	7.168278	0.169861	9.152891
C	5.817286	−8.793336	2.545847
C	5.817961	2.545821	8.793508
C	7.167264	−9.152914	0.169629
C	5.816636	−2.545696	−8.793447
C	5.817914	0.169936	9.153013
C	7.167457	−8.793375	2.545742
C	5.816664	−0.169632	−9.152977
C	5.817074	−9.152875	0.16974
C	7.166906	−2.545711	−8.793541
C	7.168491	2.545742	8.793378
C	−0.774004	4.081225	3.847579
C	0.924369	−6.249751	−3.768095
C	−11.910058	−4.881569	−2.758857
C	13.75898	−4.88229	−2.760715
C	−13.608474	5.449478	4.856667
C	12.060546	5.448743	4.854862
C	−11.90929	4.882998	2.76143
C	13.759643	4.882066	2.759623
C	0.925274	6.25024	3.768842
C	−0.774744	−4.080622	−3.846611
C	−13.609462	−5.448188	−4.853884
C	12.059577	−5.448862	−4.855719
C	−5.667125	−7.784744	1.178734
C	−7.015591	1.179139	7.785385
C	−5.667003	7.78551	−1.177116
C	−7.01773	−1.177653	−7.784209
C	−5.666213	1.537969	10.162102
C	−7.016996	10.161736	−1.536618
C	−5.667741	−1.537119	−10.160444
C	−7.017338	−10.1608	1.538466
C	−11.910081	−4.080399	−3.845749

C	13.758918	−4.081125	−3.847582
C	−13.60853	6.250623	3.769845
C	12.060497	6.24992	3.767981
C	−0.774222	4.882361	2.760713
C	0.92431	−5.448584	−4.854892
C	−0.774854	−4.881838	−2.759755
C	−13.609407	−6.249333	−3.767063
C	12.059631	−6.250065	−3.768775
C	−11.909409	4.081521	3.848283
C	13.759676	4.080884	3.84652
C	0.925327	5.44912	4.855601
C	−7.017015	7.785615	−1.177071
C	−5.667531	−1.177692	−7.784335
C	−7.017299	−7.784713	1.178846
C	−5.666471	1.178834	7.785879
C	−7.017931	−1.537082	−10.160349
C	−5.667148	−10.160838	1.538369
C	−7.016403	1.538005	10.162199
C	−5.666806	10.161698	−1.536714
C	−11.909495	−3.846361	4.082285
C	13.759475	−3.847179	4.080441
C	−0.774045	−2.759732	4.882698
C	−0.774564	2.76028	−4.88177
C	0.924581	4.855514	−5.448503
C	0.925079	−4.855019	5.449223
C	−13.608629	−3.76781	6.251372
C	12.060396	−3.768477	6.249552
C	−0.774558	3.847143	−4.080402
C	−11.909309	−2.759787	4.882854
C	13.759583	−2.760285	4.881621
C	−0.774128	−3.846678	4.081327
C	0.925166	−3.768273	6.250332
C	−13.608717	−4.854631	5.450226
C	12.060303	−4.855354	5.448376
C	0.924512	3.768811	−6.249593
C	7.872747	−1.357754	−8.973347
C	7.874105	1.357923	8.973065
C	5.111283	−8.973091	1.357827
C	5.112003	1.357896	8.973239
C	7.873274	−8.973166	1.357642
C	5.110732	−1.357641	−8.973149
C	0.075654	4.346323	4.919718
C	0.074783	−5.984726	−2.695709
C	−12.759658	−5.98431	−2.69479
C	12.909275	−5.985026	−2.696611
C	−12.758872	4.34668	4.920656
C	12.91015	4.345983	4.918846

C	−12.758965	5.98554	2.697443
C	12.910027	5.984826	2.695584
C	0.075465	5.985216	2.696571
C	0.074755	−4.345775	−4.918883
C	−12.759795	−4.345448	−4.918004
C	12.909196	−4.346134	−4.919852
C	−4.961232	−8.972808	1.358581
C	−7.72239	1.358193	8.974198
C	−4.960931	8.973625	−1.357001
C	−7.723739	−1.357259	−8.972248
C	−4.960406	1.358152	8.974002
C	−7.722914	8.973701	−1.356803
C	−4.961755	−1.357352	−8.972443
C	−7.723216	−8.972726	1.358757
C	−12.759142	−4.918716	4.347416
C	12.909854	−4.919423	4.345578
C	0.075432	−2.695879	5.985393
C	0.074773	2.696367	−5.984481
C	0.07501	4.919589	−4.345668
C	0.075349	−4.919064	4.346515
C	−12.758928	−2.695514	5.986286
C	12.910022	−2.696162	5.984419
H	−12.759363	11.733443	−5.100069
H	12.909637	11.732735	−5.101896
H	−11.324592	3.890872	−3.320852
H	14.34445	3.890101	−3.322651
H	−11.324632	2.023043	−4.697439
H	14.344292	2.022264	−4.69953
H	−14.194615	3.72587	−7.007502
H	11.474383	3.725174	−7.009326
H	−14.194465	5.593715	−5.630628
H	11.474534	5.593005	−5.632455
H	7.652925	9.276882	0.648545
H	7.652551	8.66967	−3.36437
H	5.332524	9.27694	0.648707
H	5.332218	8.669738	−3.364154
H	−12.758455	−1.401207	12.71818
H	12.910543	−1.401914	12.716357
H	−12.760188	1.402454	−12.715518
H	12.908813	1.401746	−12.717344
H	0.074313	1.402096	−12.716431
H	0.075136	11.733088	−5.100983
H	0.076044	−1.401561	12.71727
H	−12.759281	−11.732193	5.102736
H	12.909716	−11.732906	5.100912
H	0.075216	−11.732552	5.101825
H	−12.758874	12.717473	1.403163

H	12.910126	12.716765	1.401337
H	0.074204	−5.101137	−11.732402
H	0.076152	5.101673	11.733242
H	−12.759772	−12.716222	−1.400495
H	12.909226	−12.716935	−1.402321
H	−12.758347	5.102026	11.734151
H	12.910652	5.101318	11.732328
H	0.074725	−12.716582	−1.401408
H	0.075627	12.717119	1.40225
H	−12.760295	−5.100779	−11.731489
H	12.908704	−5.101487	−11.733315
H	7.652019	0.648702	−9.276937
H	7.653232	−0.648623	9.276709
H	5.332178	−8.669497	3.364226
H	5.332954	3.364338	8.669656
H	7.652373	−9.276757	−0.64875
H	5.331486	−3.364065	−8.669576
H	5.332887	−0.648549	9.276865
H	7.652667	−8.669558	3.364064
H	5.331558	0.648767	−9.276769
H	5.331896	−9.276692	−0.648598
H	7.65191	−3.364153	−8.669736
H	7.653201	3.364345	8.669486
H	−1.359415	3.322466	3.890759
H	1.509616	−7.008708	−3.724885
H	−11.324653	−4.698314	−2.021152
H	14.344306	−4.699036	−2.022998
H	−14.193669	5.632723	5.594483
H	11.475322	5.632039	5.592648
H	−11.324203	4.699255	2.023654
H	14.344849	4.698796	2.021803
H	1.510523	7.009171	3.725504
H	−1.360243	−3.321897	−3.889722
H	−14.194772	−5.631401	−5.591619
H	11.474217	−5.632128	−5.593431
H	−5.181928	−6.966391	1.055023
H	−7.503065	1.053973	6.968954
H	−5.180971	6.967009	−1.05338
H	−7.502897	−1.053616	−6.965939
H	−5.181007	1.661784	10.980425
H	−7.502126	10.980105	−1.660412
H	−5.182659	−1.660961	−10.978836
H	−7.502496	−10.979144	1.662331
H	−11.324791	−3.321517	−3.889002
H	14.34421	−3.322183	−3.890811
H	−14.193765	7.009596	3.72664
H	11.475228	7.008883	3.724843

H	−1.359595	4.699296	2.022185
H	1.509527	−5.631835	−5.592727
H	−1.3598	−4.69853	−2.021752
H	−14.194678	−7.008273	−3.723774
H	11.474294	−7.008964	−3.725675
H	−11.323883	3.322754	3.891423
H	14.344962	3.321941	3.889588
H	1.510621	5.632264	5.593383
H	−7.502231	6.967288	−1.053243
H	−5.182394	−1.05366	−6.966019
H	−7.502445	−6.966335	1.055263
H	−5.181162	1.054275	6.967685
H	−7.503137	−1.660898	−10.97867
H	−5.182018	−10.979208	1.662164
H	−7.501485	1.661848	10.98059
H	−5.181648	10.980041	−1.660578
H	−11.324374	−3.889732	3.323289
H	14.344723	−3.89031	3.321472
H	−1.359564	−2.022077	4.699298
H	−1.360138	2.022819	−4.698267
H	1.509814	5.593297	−5.631722
H	1.510314	−5.592833	5.632359
H	−14.193825	−3.724547	7.010371
H	11.475167	−3.725278	7.008541
H	−1.360264	3.890959	−3.321148
H	−11.324251	−2.021445	4.700712
H	14.344855	−2.022524	4.698328
H	−1.359773	−3.889947	3.322719
H	1.510462	−3.724945	7.00926
H	−14.193977	−5.59239	5.633497
H	11.475015	−5.593083	5.631694
H	1.509649	3.725378	−7.008636
H	−17.410822	−1.357023	−8.971554
H	−17.409998	8.973968	−1.356107
H	−17.410299	−8.972464	1.359439
H	−17.409473	1.358523	8.974882
H	17.560351	−8.973432	1.35695
H	17.561178	1.357559	8.972394
H	17.559829	−1.357986	−8.974042
H	17.560654	8.973002	−1.3586

Table S6. The positions of 64 adsorbed H_2.

Atom	x (Å)	y (Å)	z (Å)
H	1.507513	0.922868	6.141642
H	2.15933	1.006601	6.48795
H	1.504705	−6.139441	0.923518
H	2.155721	−6.486868	1.008917
H	1.51085	−0.922059	−6.142105
H	2.161186	−1.006678	−6.490955
H	1.505168	6.141943	−0.924004
H	2.156394	6.488936	−1.009713
H	11.491867	6.154601	−0.929603
H	10.837542	6.491158	−1.032437
H	11.491523	−0.929736	−6.15738
H	10.836711	−1.034361	−6.492397
H	11.492211	0.929321	6.154087
H	10.837859	1.032369	6.490532
H	11.491867	−6.157464	0.929521
H	10.837001	−6.492338	1.034255
H	−3.039862	0.951888	5.184552
H	−3.358667	0.840622	4.526163
H	−2.959785	−1.008756	−5.114768
H	−3.278523	−0.895742	−4.456676
H	−3.028709	−5.150757	1.107359
H	−3.345069	−4.492142	0.990202
H	−2.97157	5.151917	−1.021366
H	−3.286194	4.491658	−0.909528
H	−9.638207	−1.016149	−5.211745
H	−9.321912	−0.902205	−4.552665
H	−9.63277	5.182101	−1.035654
H	−9.316956	4.523276	−0.918519
H	−9.851674	1.204217	5.040166
H	−9.519057	1.066909	4.393895
H	−9.714628	−5.152339	0.996249
H	−9.397862	−4.49253	0.887532
H	−0.279775	−4.159522	0.461973
H	−0.165125	−3.434127	0.562452
H	−0.288459	0.458405	4.123574
H	−0.251916	0.545909	3.389003
H	−0.231395	4.148511	−0.447797
H	−0.09805	3.426113	−0.54653
H	−0.183561	−0.424488	−4.161052
H	−0.028295	−0.521421	−3.443009
H	−12.316108	4.120186	−0.470967
H	−12.377156	3.387567	−0.56124
H	−12.464295	0.453897	4.033248
H	−12.619853	0.534782	3.313802
H	−12.462814	−4.126389	0.405427

H	−12.61389	−3.406231	0.491096
H	−12.289675	−0.482381	−4.059832
H	−12.26779	−0.56419	−3.324325
H	−3.319398	1.341151	−6.926241
H	−3.651	1.997196	−7.017865
H	−3.17637	−3.625056	−6.872586
H	−3.294209	−4.349224	−6.770208
H	−3.182025	3.622629	6.866368
H	−3.299304	4.346791	6.763233
H	−3.327721	−1.363213	6.975565
H	−3.657154	−2.019622	7.071359
H	−3.317951	6.940526	1.358725
H	−3.648718	7.032179	2.015141
H	−3.178984	6.87113	−3.622638
H	−3.296781	6.76775	−4.34666
H	−3.167351	−6.89076	3.634651
H	−3.284899	−6.789667	4.359035
H	−3.327245	−6.924807	−1.346363
H	−3.659656	−7.013451	−2.002395
H	−9.361273	−6.885784	−1.338978
H	−9.027942	−6.978754	−1.99385
H	−9.444394	−6.766811	3.614311
H	−9.329693	−6.656211	4.337696
H	−9.118037	−1.236725	6.443262
H	−8.686076	−1.693157	6.052042
H	−9.161779	3.52728	6.570653
H	−8.666367	4.053514	6.412424
H	−9.35187	6.922279	1.349734
H	−9.022387	7.014743	2.006601
H	−9.426935	6.757387	−3.626314
H	−9.30565	6.648263	−4.348598
H	−9.34967	1.355991	−6.954465
H	−9.021518	2.012969	−7.050504
H	−9.42898	−3.62913	−6.759708
H	−9.309986	−4.351787	−6.650446
H	11.018879	2.710259	−3.596365
H	10.513694	2.414975	−3.144341
H	6.409448	6.323023	−0.959496
H	6.346626	5.596943	−0.833354
H	6.434992	−0.958995	−6.320937
H	6.392834	−0.832711	−5.593504
H	6.392546	0.959769	6.324209
H	6.315365	0.833744	5.599408
H	6.457375	−6.314596	0.957805
H	6.431801	−5.586486	0.831426
H	1.894702	−3.550059	−2.669411
H	2.374885	−3.079087	−2.361858

H	11.021882	−3.577239	−2.741333
H	10.516306	−3.110954	−2.469625
H	11.015763	3.575904	2.734192
H	10.506855	3.113962	2.461375
H	1.896156	3.564306	2.64683
H	2.374702	3.100796	2.325806
H	1.885011	2.614257	−3.574297
H	2.355449	2.268675	−3.120231
H	11.015664	−2.733862	3.576107
H	10.506777	−2.460243	3.114621
H	1.892176	−2.638337	3.566983
H	2.368022	−2.307292	3.107751
H	−10.287192	1.448801	−2.04113
H	−9.675745	1.747683	−2.329469
H	−10.358019	2.023894	1.361855
H	−9.744701	2.325056	1.643567
H	−6.529901	0.18728	4.51649
H	−6.406553	0.903788	4.382621
H	−6.269953	4.32891	−1.214356
H	−6.403907	4.451634	−0.497921
H	−9.509771	−2.440291	−1.78056
H	−10.115653	−2.137174	−1.485235
H	−3.220228	2.529822	1.841099
H	−2.781297	2.117612	1.412542
H	−2.845317	−2.178217	−1.370496
H	−3.281535	−2.599688	−1.792164
H	−6.348997	−1.240858	−4.312659
H	−6.229395	−0.521712	−4.434914
H	−6.31115	−4.449059	0.317249
H	−6.449991	−4.328462	1.033112
H	−9.176975	−1.907471	2.876341
H	−9.604836	−1.489676	2.442126
H	−2.270777	1.361245	−1.96388
H	−2.884707	1.645404	−2.261639
H	−2.350038	−1.392099	1.99239
H	−2.968134	−1.672824	2.28473

References

1. Sillar, K.; Hofmann, A.; Sauer, J. Ab Initio Study of Hydrogen Adsorption in MOF-5. J. Am. Chem. Soc. 2009, 131, 4143–4150
2. Klontzas, E.; Mavrandonakis, A.; Froudakis, G. E.; Carissan, Y.; Klopper, W. Molecular Hydrogen Interaction with IRMOF-1: A Multiscale Theoretical Study. J. Phys. Chem. C 2007, 111, 13635–13640
